# Using systems science to understand the determinants of inequities in healthy eating

**DOI:** 10.1371/journal.pone.0188872

**Published:** 2017-11-30

**Authors:** Sharon Friel, Melanie Pescud, Eleanor Malbon, Amanda Lee, Robert Carter, Joanne Greenfield, Megan Cobcroft, Jane Potter, Lucie Rychetnik, Beth Meertens

**Affiliations:** 1 School of Regulation and Global Governance (RegNet), Australian National University, Canberra, Australia; 2 Sax Institute, Sydney, Australia; 3 Deakin University, Melbourne, Australia; 4 ACT Health, Canberra, Australia; 5 NSW Health, Sydney, Australia; 6 National Heart Foundation, Melbourne, Australia; University of Bremen, GERMANY

## Abstract

**Introduction:**

Systems thinking has emerged in recent years as a promising approach to understanding and acting on the prevention and amelioration of non-communicable disease. However, the evidence on inequities in non-communicable diseases and their risks factors, particularly diet, has not been examined from a systems perspective. We report on an approach to developing a system oriented policy actor perspective on the multiple causes of inequities in healthy eating.

**Methods:**

Collaborative conceptual modelling workshops were held in 2015 with an expert group of representatives from government, non-government health organisations and academia in Australia. The expert group built a systems model using a system dynamics theoretical perspective. The model developed from individual mind maps to pair blended maps, before being finalised as a causal loop diagram.

**Results:**

The work of the expert stakeholders generated a comprehensive causal loop diagram of the determinants of inequity in healthy eating (the HE^2^ Diagram). This complex dynamic system has seven sub-systems: (1) food supply and environment; (2) transport; (3) housing and the built environment; (4) employment; (5) social protection; (6) health literacy; and (7) food preferences.

**Discussion:**

The HE^2^ causal loop diagram illustrates the complexity of determinants of inequities in healthy eating. This approach, both the process of construction and the final visualisation, can provide the basis for planning the prevention and amelioration of inequities in healthy eating that engages with multiple levels of causes and existing policies and programs.

## Introduction

Systems thinking has emerged in recent years as a promising approach to understanding and acting on the prevention and amelioration of non-communicable disease (NCDs). The interest in systems thinking arises from the growing body of evidence acknowledging the multiple, systemic and complex causes of NCDs; and that to address them, actions are required at the individual and societal level [1–5]. The lack of explicit attention to the function of the system as a whole has led public health professionals, policy makers and researchers to call increasingly for more systems oriented analyses for public health issues [2, 6–13]. For example, the UK Foresight study worked across sectoral boundaries to create a systems diagram of the variables that contribute to obesity [14]. A recent study from Allender *et al*. demonstrated the effectiveness of using systems methods to identify the determinants of childhood obesity within local communities [15]. However the evidence and perspectives on *inequities* in NCDs and their risks factors, particularly diet, has not been examined from a systems perspective.

An unhealthy diet is one of the top risk factors for cardiovascular disease, Type 2 diabetes, certain cancers, and osteoporosis [16–18]. There has been a large global shift towards diets of highly refined and often ultra-processed foods, and higher intakes of meat and dairy products [19, 20]. This transition has been accompanied by large numbers of people consuming excess energy, contributing to overweight and obesity in more than two billion people in 2013 [21]. These dietary health risks are not experienced equally. In high and middle income countries, people who are socially disadvantaged are more likely to eat unhealthy diets and have higher levels of nutrition-related disease compared to people further up the social hierarchy [5, 22–25].

Research and policy approaches to diet-related health issues have, to date, given primary emphasis to the role of individuals and their knowledge, preferences and behaviours [26]. A more recent socio-ecological conceptualisation of dietary behaviours and inequities in healthy eating has identified many important factors at the societal level that shape individual preferences and behaviours [27–30]. The socio-ecological model posits that the systematic evolution, continuation and, occasionally, improvement in the social distribution of dietary intake and related health outcomes illustrates the influence of broader societal issues on daily practices, such that people’s ability to pursue healthy behaviour is compromised with decreasing social status. What, where and how much people eat are responses to their economic, environmental and cultural contexts [5, 31–34]. The limitation of the socio-ecological approach is that it does not explicitly capture the interactions between the different factors that influence inequities in healthy eating.

Systems science helps organise and analyse complex information with an emphasis on the whole picture and the interactions between variables. Systems approaches are thus well suited to understanding public health issues, including inequities in healthy eating [35–37]. Systems science considers an observed phenomenon, such as inequities in healthy eating, to be part of, or emerging from, the structure of complex adaptive systems. A ‘complex adaptive system’ refers to a collection of elements (e.g. sub-systems, sectors) and the interconnections between those elements that give rise to dynamic behaviour [37–39]. The adaptive part of a complex adaptive system refers to the ability for a system to change, e.g. in response to external pressures [40]. The value of calling an observed situation a ‘system’ is to emphasize that it is not possible to understand the phenomena by studying its parts or elements in isolation; attention to the dynamics between the parts is fundamental [37]. It is important to note that properties that emerge from systems that involve humans do not do so automatically or passively; rather the use of the term “emerge” draws attention to the human decisions, including policies, that have caused a system to be structured in a way that gives rise to certain outcomes, such as inequities in dietary behaviours. Unlike the socio-ecological conceptual approach, a systems approach also enables consideration of feedback loops within the system that drive inequities in healthy eating [27].

While some of the links between specific aspects of the food system, social and individual level factors have been studied in detail, the interconnections between these factors and the effects of the whole system on inequities in healthy eating have not been investigated. This study sought to organise, from a complex systems perspective, current understandings of how individual and societal level factors interact to create inequities in healthy eating. It also sought to identify policy relevant factors and their dynamic interactions. This was achieved using an established method in system science, collaborative conceptual modelling (CCM) [41], to develop a causal-loop diagram that is representative of a complex adaptive system. The purpose of doing this was to understand the whole system (hereafter referred to as the HE^2^ system, with the H referring to ‘healthy’ and the E^2^ to ‘equitable’ and ‘eating’) and to begin to identify key leverage points to more effectively address inequities in healthy eating.

In this paper we present the results of research conducted with an expert group of policy actors (policy makers, practitioners and researchers), which is part of a larger study looking at policy development and implementation challenges associated with action on inequities in healthy eating. The larger study seeks to answer the overarching question ‘What kind of insight can policy actors gain about causes of, and solutions to, inequities in healthy eating using systems science methods?’

## Methods

This study used qualitative methods. We have followed the Consolidated Criteria for Reporting Qualitative Research (COREQ) checklist for reporting qualitative research [42]. Participants provided written informed consent to participate in this study. The study received ethics clearance from the Australian National University Human Research Ethics Committee, reference number 2014/343 ‘HE2: A systems approach to healthy and equitable eating’.

### Research team and reflexivity

The study was designed and conducted by a core team (SF, EM, MP) who consulted with a larger project team consisting of public health academics and health practitioners through the design, data collection and analysis steps. The disciplinary backgrounds of the core team comprised systems science, public health and epidemiology and population nutrition. EM facilitated the CCM work and digitalised the HE^2^ system.

### Study design

Systems methods can be both qualitative and quantitative in nature, including the formation of dynamic systems models, causal loop diagrams, agent-based models, network analyses and time series analyses [37]. In this study we use a qualitative soft systems method, based on the principles of system dynamics known as ‘Collaborative Conceptual Modelling’ (CCM), to create a causal loop diagram relating to inequities in healthy eating [41]. The method enabled the integration of the knowledge and experience of key stakeholders/experts from different sectors about the drivers of inequities in healthy eating and identify relationships, feedback loops and possible unintended consequences. We describe below the steps followed in the construction of the diagram according to the CCM method.

#### Selection of key stakeholders / policy actor experts

The HE^2^ system and development of the causal loop diagram was informed by a group of policy experts working in government, non-government health organisations and academics in Australia, who were united by a concern for reducing inequities in healthy eating. The experts were linked by their involvement in The Australian Prevention Partnership Centre (TAPPC)—a national initiative supporting the prevention of lifestyle related chronic disease through the co-production of knowledge by academics, health system practitioners and federal and state-level policy makers [43]. The experts were recruited through the TAPPC network and were selected based on their expertise in the issue and availability for participation.

As outlined in the CCM method [41], we used an inclusive definition of ‘expert’ to refer to a person who has observed and thought seriously about how parts of the system relate to inequities in healthy eating. The disciplinary backgrounds of the expert group included public health and epidemiology; population nutrition; health economics; social marketing, and public policy. This disciplinary diversity strengthened the likelihood of capturing a range of perspectives in the systems diagram. Due to the challenges of creating causal loop diagrams with large numbers of people [41], the expert group was limited to 12 people plus three members of the core research team. This number of participants served to promote interaction and discussion between all participants. It also facilitated effective communication between members, with a balance of voices and perspectives being heard throughout the process of creating the diagram. The professional roles of the participants are described in Table 1. The potential perspectives represented in the group were not exhaustive—they did not include, for example, food retailers or manufacturers, food service providers (although two of the members previously worked in the food industry) or the general public/consumers.

**Table 1:** Expert group participants’ professional roles.

Institution	Role
University, Research Organisation, and or Medical Practice	Academic, public health nutrition
	Academic, health economics
	Academic, public health nutrition, health policy
	Academic, social determinants of health, policy
	Academic, public health nutrition, health policy
	Academic, systems modeller
	Academic, health policy and practice
	Academic, child obesity
	Communications officer
Private consultant	Systems expertise
State government	Policy officer, health promotion and public health nutrition
	Policy officer, food policy
	Policy officer, food policy
Non-government organisation	Policy practitioner, food and public health nutrition policy
	Policy practitioner, health equity policy

#### Data collection and analysis

Data were collected and analysed over two three-hour participatory workshops–the first in May 2015 and the second in November 2015. Both workshops were held at the Australian National University in Canberra and were facilitated by EM.

In the May workshop, participants received an overview of the project and an explanation of system science approaches to the study of complex problems such as inequities in healthy eating. The experts were asked to: i) discuss the problem of inequities in healthy eating and the various factors/variables that affect it; and ii) develop their individual ‘mental models’ and combined ‘pair blended’ models of connections between variables, and the nature of the relationships between variables (see Table 2). The construction of individual mental models followed by pair blending is a distinguishing feature of the CCM method, which allows for increasing levels of collaboration as the method progresses.

**Table 2:** Instructions used to guide workshop participants.

Activity	Aspects Covered
Introduction	- Goals and purpose of the systems thinking approach - Explanation of what a systems diagram is (Foresight example) - Explanation of process: we will ‘collaboratively build’ our diagrams by starting individually and incrementally blending them together
Phrasing variables	- Introduction to correct phrasing of variables - Worksheet: Examples such as ‘level of…’, ‘number of…’
Level of detail required	- Introduction to correct level of detail using Foresight examples
Arrows of influence	- Introduction to the meaning of ‘influence’ and correct use of arrows
Boundaries and focus	- Explaining that we will focus on the dynamics that contribute to the equitable distribution of healthy eating
Introduction of the central mechanism	- Central stock and flow section in which the two stocks are the degree of ‘inequitable distribution of healthy eating’ and the degree of ‘equitable distribution of healthy eating’ across a population
Drawing individual influence diagrams	- Participants starting to make influence diagrams
Pair blending	- Taking a moment to digest partner’s diagram: ask any questions - Merging diagrams attempting to establish links between individual variables - Considering: What new causal connections should we draw? What else is missing?
Presenting diagrams and troubleshooting	- Troubleshooting diagrams: Can variables be phrased better? Can linkages be expanded to give better detail? - Each pair presenting back to the group, sharing any observations - Group considers ‘what else’ could be added for each diagram
Assessment of what we have	- Considering the diagrams together - Asking the group: What has been missed? What needs further elaboration? What can be synthesised? What is most important?
Building on the current work	- Dividing into two groups and blending the four diagrams into two pair-blended diagrams, continuing to combine models until a single (extensive) mind map is achieved

#### Individual mental models

Participants were required to first independently draw diagrams of factors that influence inequities in healthy eating. The creation of individual mental models allowed for all individual ideas and perspectives to emerge that may have been lost if the group worked together from the beginning.

#### Pair blending

After the initial individual mental models were drawn, participants were paired together and each pair were invited to combine their two individual diagrams into one. The group then used the pair-blended diagrams to continue to combine models until a single (extensive) mind map was achieved of the group’s understanding of the influence and interactions between different variables in the HE^2^ system.

After the first workshop, the systems modeller (EM) transferred all of the variables from the individual, pair-blended, and combined group diagrams into Vensim [44], (which is a software package for building a variety of simulation models, including causal loop diagrams) and created the initial HE^2^ causal loop diagram (CLD). The core team reviewed the workshop notes and the mind maps, checking that all of the connections identified in the workshop were captured in the CLD. Groupings of variables were identified and organized according to emergent themes (sub-systems) by members of the core team. Any changes made to the variable names or connections generated in the first workshop were noted for discussion with the experts at the second planned workshop.

The same group of experts were invited to reconvene at a second workshop in Canberra in November 2015. At this workshop, participants were reminded of the mind maps that they created in the first workshop in May. The purpose of this workshop was to review the CLD, check that the discussions from the first workshop were properly captured in the CLD and fill in any gaps. The core research team explained the process they had used to create the initial CLD derived from workshop 1, which was then presented to the group. The experts were asked to review the CLD, add any other variables or connections to the diagram, and correct any mistakes or misrepresentations in the way individual and pair blended maps were reflected. They were also asked to check that the polarity of each relationship was described correctly—as either positive or negative (i.e. a positive polarity refers to one where variables change in the same direction and a negative polarity where they change in opposite directions). Following the second workshop, the core team further refined the CLD. Variable names, direction of linkages and polarity were discussed, and the variable groupings into emerging sub-systems were modified to reflect the perspectives of the expert group.

## Results

**Fig 1:**
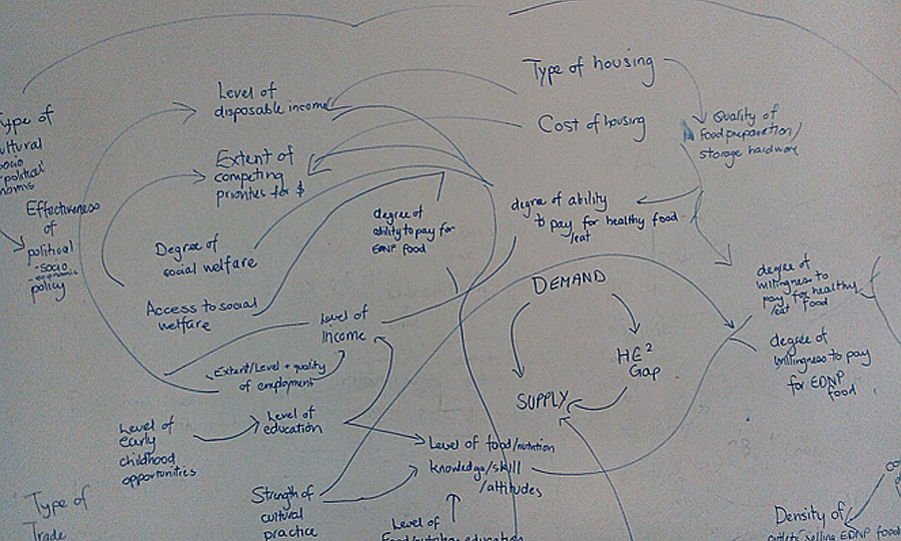
An example of a policy actor mind map of the determinants of inequities in healthy eating.

The group of experts identified multiple determinants of inequities in healthy eating. These were at multiple levels and interconnected. For example, the group identified people’s food preferences, factors in the food environment such as food price, issues of housing type, and a range of other policy issues, including social welfare and planning. Pictures of the mind maps as they were emerging during the first workshop are shown in Fig 1.

### The HE^2^ causal loop diagram

**Fig 2:**
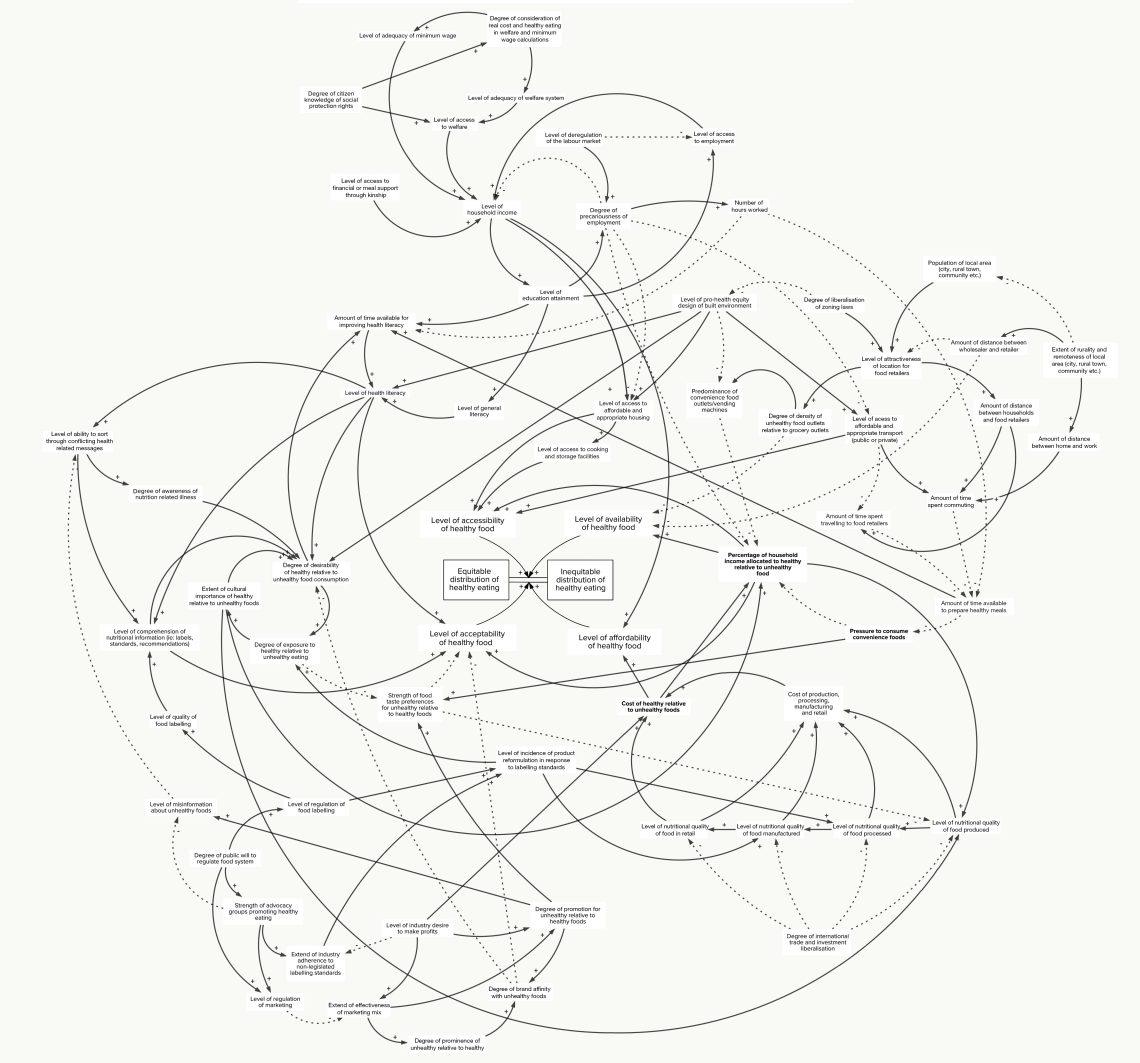
HE^2^ causal loop diagram of the determinants of inequities in healthy eating. The HE^2^ diagram is structured according to accepted principles of system dynamics [48]. The arrows indicate the direction and polarity of influence. The solid lines indicate positive polarity and the dashed lines indicate negative polarity. Positive polarity means that the initiating variable influences the receiving variable in the same direction of change (e.g. as the ‘distance between households and food retailers’ goes up, so does the ‘time spent travelling to food retailers’). Negative polarity means that the initiating variable influences the receiving variable in the opposite direction (e.g. when the ‘level of misinformation about unhealthy foods’ falls, an individual’s ‘ability to sort through conflicting health related messages’ rises). Polarities do not indicate the rate of influence, and it is important to note that change may occur at uneven rates within the diagram.

After much clarification of variables, relationships between variables and direction of connections, via email and telephone following the first workshop, and after review at the second workshop, the final CLD was completed. The CLD (Fig 2) is referred to as the ‘HE^2^ diagram. The HE^2^ diagram sets out a representation of the determinants of the social distribution of healthy eating according to the knowledge and experience of the expert group.

The decisions about which variables are included and excluded from a causal loop or other depiction of a system amount to the boundaries of the system [45]. The boundaries of the HE^2^ system were set by the expert group as they chose variables for inclusion (and implicitly, exclusion) in the workshops and any subsequent online discussions. According to the research team’s goal of identifying public policy relevant determinants of inequities in healthy eating, the desired focus of the causal loop diagram was to identify policy relevant factors and their dynamic interactions. Based on this, the group opted to be inclusive of many determinants and the feedback loops that propel them, rather than to depict fewer determinants in finer detail. The selection of included variables was based on the groups’ expert knowledge in the fields of food security, public health nutrition, health economics and the social determinants of health, informed by a substantial body of work that has been carried out in recent years with respect to the food system and the food environment, and also other policy areas that have been identified as important for achieving equity in healthy eating [5, 46, 47].

When constructing the diagrams, members of the expert group began to identify reinforcing feedback loops. Feedback is a circular chain of influence that can act to perpetuate or reinforce the state of the system variables. A change in one variable can trigger a string of connections that result in either amplifying or reducing the original variable. The change ‘feeds back’ into itself. Most variables within the HE^2^ diagram are part of at least 50 feedback loops. The HE^2^ diagram depicts a highly interconnected system, demonstrating that experts within the field are acutely aware of the connections between factors that influence inequities in healthy eating.

### The system core

**Fig 3:**
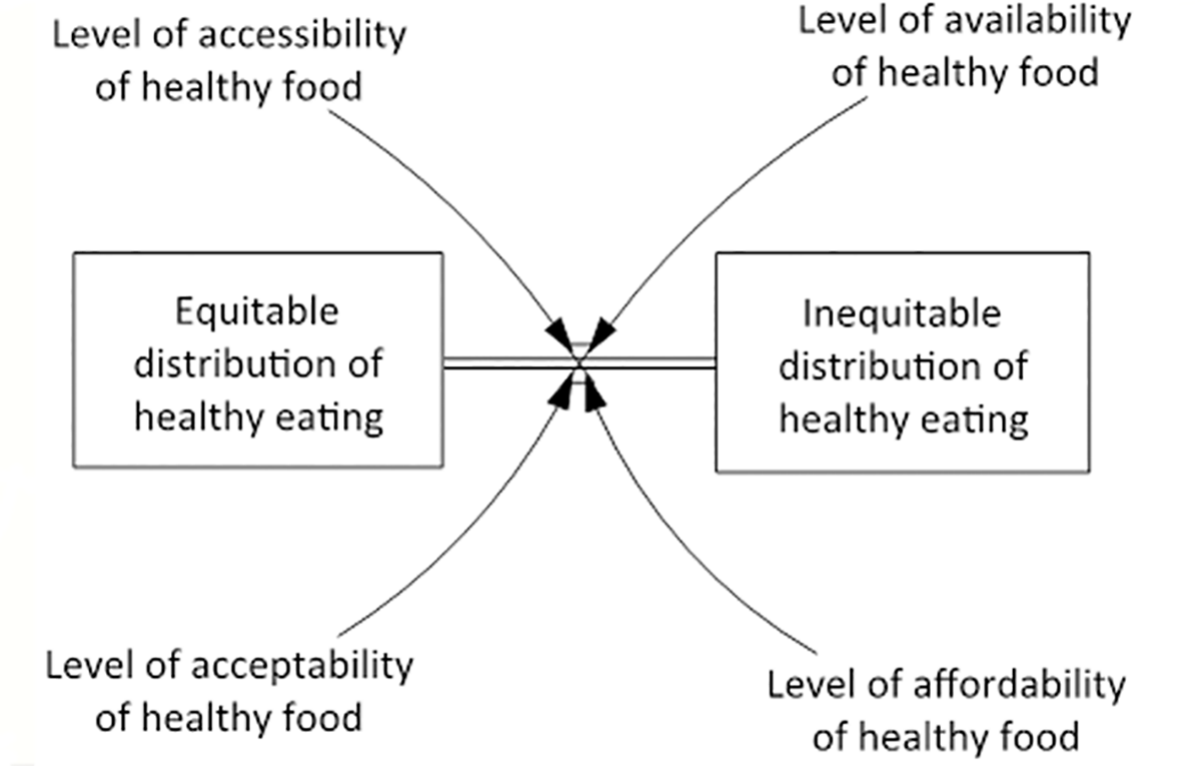
Core mechanism of the HE^2^ diagram.

At the core of the HE^2^ diagram is the central stock and flow section (depicted separately in Fig 3), in which the two stocks are the degree of ‘inequitable distribution of healthy eating’ and the degree of ‘equitable distribution of healthy eating’ across a population. The terms ‘stock and flow’ refers to the accumulation of a variable over time (stock) and the rate of change between variables (flow). The arrow between these indicates the rate of change, and the variables that influence this rate of change are the acceptability, affordability, accessibility and availability of healthy food (which are in turn influenced by all of the variables captured in the wider causal loop diagram). This central structure allows the dynamic shift from an inequitable distribution in healthy eating towards a more equitable distribution to be captured in the diagram. A draft stock and flow system core was developed by the expert modeller prior to the first workshop. This was amended throughout the two workshops while making the CLD (i.e. both the CLD and system core were developed using an iterative process). While stocks and flows are often quantified, the expert group and the resulting HE^2^ CLD focused on first articulating the systems structure of equity/inequity in healthy eating, and the unnumbered stocks are adequate for this purpose. The *a priori* hypothesis that gave rise to the system core is that for the level of equity in healthy eating to rise there needs to be greater consumption of a healthy diet by all Australians and also a proportionally greater percentage increase among socially disadvantaged groups.

### Sub-systems

**Fig 4:**
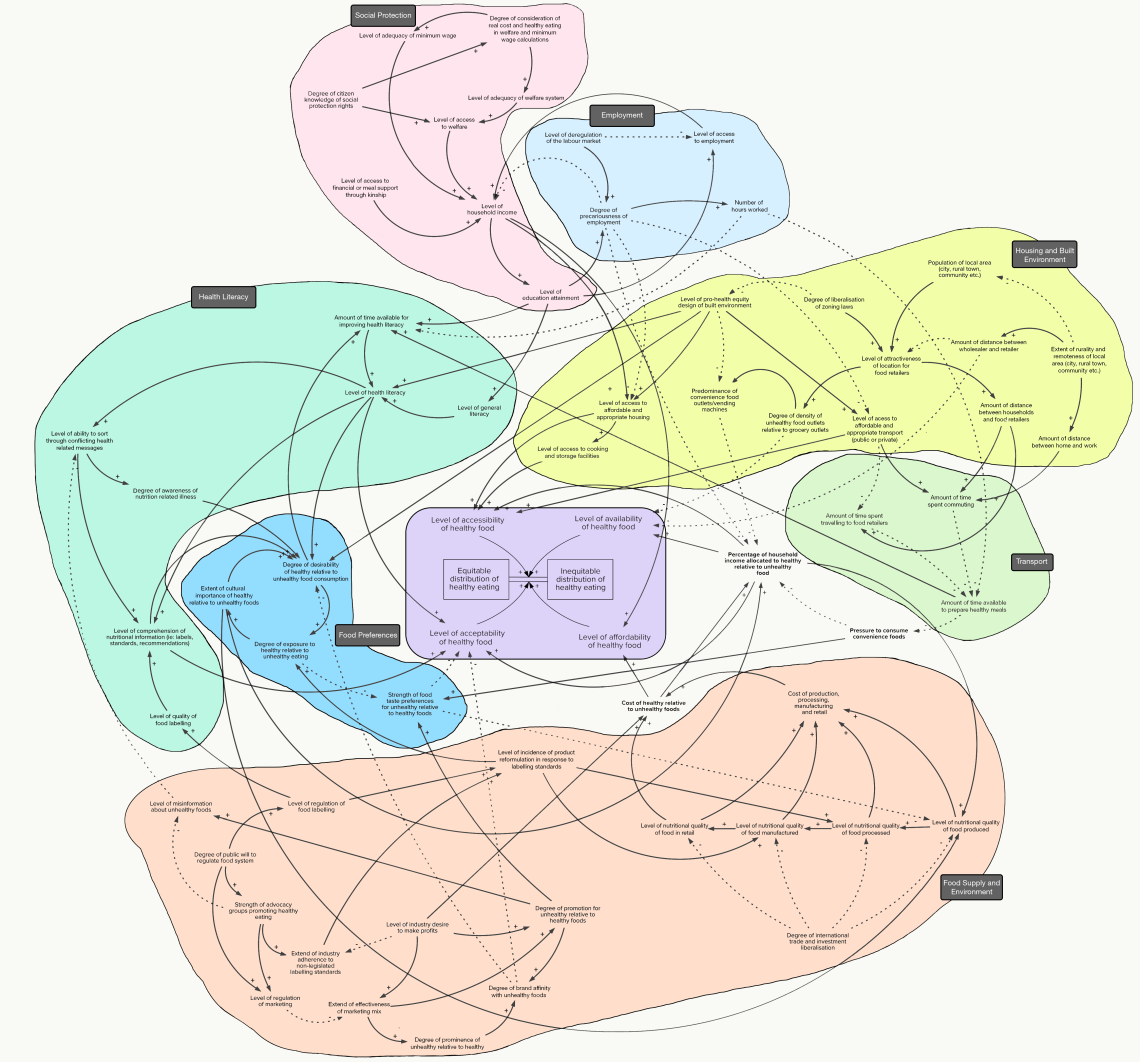
CLD for determinants of inequities in healthy eating, showing sub-systems. The arrows indicate the direction and polarity of influence. Solid lines indicate positive polarity and dashed lines indicate negative polarity. Positive polarity means that the initiating variable influences the receiving variable in the same direction of change (e.g. as the ‘distance between households and food retailers’ goes up, so does the ‘time spent travelling to food retailers’). Negative polarity means that the initiating variable influences the receiving variable in the opposite direction (e.g. when the ‘level of misinformation about unhealthy foods’ falls, an individual’s ‘ability to sort through conflicting health related messages’ rises). Polarities do not indicate the rate of influence, and it is important to note that change may occur at uneven rates within the diagram.

The HE^2^ diagram comprises 67 variables and 129 connecting arrows, highlighting the perception among the expert group of a high degree of complexity, interconnectedness and feedback between variables in the system. Most variables within the HE^2^ diagram are part of at least 50 feedback loops. Various groupings of variables–sub-systems–emerged through the workshops. As shown in Fig 4, each variable was allocated into a sub-system relevant to macro-level policy domains such as ‘transport’ and ‘social protection’, and also more meso/micro level factors such as ‘health literacy’ and ‘food preferences’. There were one or two factors raised during the workshop that did not warrant sub-systems of their own and did not quite fit into the other sub-systems. Trade and investment liberalization was the main one, and was recognised by the group as influencing the availability, affordability and acceptability of different foodstuffs, as well as affecting labour markets and employment conditions, with the attendant effects on healthy eating as described in those particular sub-systems.

The breakdown of the diagram into seven sub-systems also serves to enhance communication of the diagram and the ways in which the variables related to inequity in healthy eating span sectoral boundaries—of the 129 connecting arrows, 48 illustrate connection between the sub-systems. The goal of the study was to take a ‘high level’ look at the whole system. It is therefore impractical to describe each linkage and variable in the HE^2^ diagram. Instead, the following discussion highlights the key variables identified within the main sub-systems by the expert group.

#### Food supply and environment

Much of the discussion at the workshops focused on issues to do with food supply and the food environments. This is depicted as the Food Supply and Environment sub-system, which illustrates the movement of nutrition quality, access and price through each stage of the food environment from production to processing, manufacturing, and ultimately availability in the retail and food service environments. Members of the expert group spoke about the various opportunities and barriers to improving equity in healthy eating through policy and actions in the food supply and environment that would improve levels of availability, accessibility, affordability and acceptability of healthy foods relative to unhealthy foods. An observed feedback loop in this sub-system is between food labelling–including the influence of advocacy and industry groups on implementation of mandatory or voluntary arrangements, respectively–and the impact labelling has on reformulation and marketing of food.

#### Housing and the built environment sub-system

The Housing and Built Environment sub-system represents the physical conditions through which people must access food, and the ways in which the conditions of the built environment, including housing, affect that access. Significant variables identified by the expert group included the degree of rurality or remoteness of an area. Rurality and remoteness, which affect the distance between wholesaler and retailer, were considered to be significant factors in the current social distribution of healthy eating in Australia—the challenges in providing fresh food to remote communities are well documented [49, 50]. The participants also identified the distance between homes and workplaces to healthy food outlets and retailers as being important to the accessibility of healthy food relative to unhealthy food. The built environment was also noted to influence market conditions, such as the attractiveness of a location to food retailers and outlets, and the prominence of convenience food outlets. Housing was another variable discussed by the group. With access to affordable and quality housing being a key factor for health in general, and healthy eating in particular–access to facilities for cooking and storing food, were considered essential conditions for maintaining a healthy diet. Affordable housing and housing quality are major issues among lower socioeconomic groups and improving them would significantly help to reduce health and dietary inequities.

#### Transport sub-system

The transport sub-system captures the way that the dynamics between distance, work and time affect inequities in healthy eating. Access to affordable transport among lower socioeconomic groups, either public or private, was noted by the expert group as influencing the time spent commuting and time spent traveling to work and other regular places of attendance. These factors were believed to go on to determine the time available to prepare healthy meals and engage in a healthy diet, and the time spent improving health literacy.

#### Employment sub-system

The group identified that work matters for inequities in healthy eating due to the affordability of a healthy diet being determined not just by the price of food, but also by the disposable income of individuals and households. Participants spoke about families or households who are unemployed or in low paid jobs finding it difficult to afford a healthy diet. Precarious employment conditions such as shift work, variable, non-standard or inflexible work hours, working overtime and multiple jobs, lack of job security, low pay, and low status jobs were all identified as being associated with fewer family meals prepared or eaten at home, poorer nutritional quality of meals, and less healthy diets. Working conditions can also shape food choices indirectly through their influence on time stress and time available for meal planning, food shopping, and preparation and their contribution to stress, fatigue and dissatisfaction. The variable ‘distance between home and work’ was placed into the employment sub-system to highlight the power that some employers have to enable work from home options for their employees, and thus reduce commuting time pressures.

#### Social protection sub-system

The Social Protection sub-system represents the formal and informal social conditions that were identified as shaping people’s opportunities to eat healthily, and were noted by the group as being strongly connected to the Employment sub-system. Important social protection factors identified include level of educational attainment, regulatory controls over the labour market, the adequacy of the welfare system and the minimum wage, which influence levels of household income and the affordability of healthy food. According to the HE^2^ Diagram, a key factor affecting the adequacy of the welfare system and of the minimum wage is the consideration of the real cost of healthy eating in the design of these social protection mechanisms. The group also acknowledged the informal supports that can help improve equity in healthy eating, including factors such as kinship and community groups, which provide financial or meals based support to people in need.

#### Health literacy sub-system

The group also identified a number of individual level variables, notably in the health literacy and food preference sub-systems. The Health Literacy sub-system represents consumers’ ability to make sense of different health messages, their degree of comprehension of nutrition information (nutrition knowledge) and the willingness and time available to improve health literacy. Nutrition knowledge was noted by the group as being a key equity concern, whereby people from low socioeconomic groups typically report lower levels of nutrition knowledge. Nutrition knowledge can act as an antecedent to healthy food choices, however many other intersecting factors often influence health literacy, as illustrated in the Health Literacy sub-system.

#### Food preferences sub-system

Food preferences were noted by participants as being important individual level factors that affect food choices. Preferences and levels of acceptance for new foods can be altered via repeat exposures to disliked or unfamiliar foods. Within the current obesogenic food environment, the group felt that it is often difficult for communities to avoid the heavily marketed highly palatable foods that are often high in fat, salt, and sugar.

### Dynamics of the HE^2^ system as a whole

**Fig 5:**
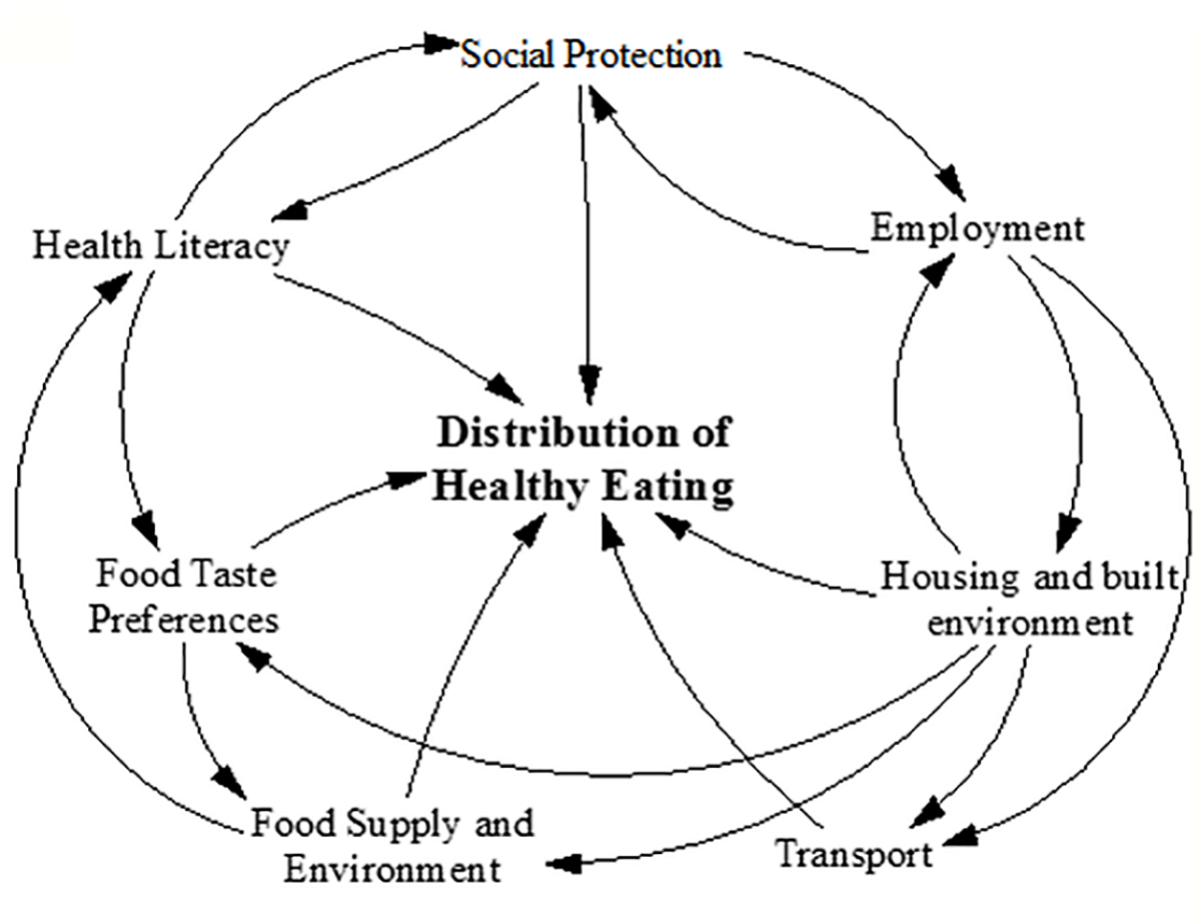
Feedback between sub-systems and the social distribution of healthy eating.

Fig 5 is a structural level depiction of the HE^2^ diagram that shows the feedback between different sub-systems as they relate to inequity in healthy eating. The arrows are indicative of the high level connections between the sub-systems, illustrating the ways in which policy domains interact to give rise to equity/inequity in healthy eating. The direction of the arrows connecting each sub-system are derived from the direction of the arrows in the full HE^2^ CLD (Fig 2). The structural level diagram gives insight into the feedback between policy sub-systems at a structural level. From this diagram it can be observed that the distribution of healthy eating is influenced by all sub-systems. Further, this diagram indicates that the state of variables within a certain sub-system, for example Housing, influence the distribution of healthy eating directly, but also go on to influence the state of variables in other sub-systems, such as Transport, Employment and, Food Supply and Environment. The state of variables in these sub-systems can then go on to influence variables in the Housing sub-system, and so on. Such feedback based interactions between sub-systems add to the complexity of the HE^2^ system overall.

## Discussion

In this paper we present the results of research conducted with an expert group of policy actors (policy makers, practitioners and researchers). A systems approach, using group model building of a causal loop diagram, has made it possible to organise and analyse complex information with an emphasis on the whole picture. While the healthy eating literature has paid a lot of attention to food systems, this project expands the thinking in two ways: it brings an equity lens to healthy eating, and it considers a much wider system that is inclusive of societal and individual level factors that cross multiple sectors—the food supply and environment; housing and the built environment; transport; employment; social protection; health literacy, and food preferences. This articulation of the interconnections within the wider system highlights the need, within policy development and implementation, to consider the ways in which actions in one sector can reinforce or undermine actions in other sectors. Members of the policy actor group were able to identify multiple level determinants of inequities in healthy eating and capture the interactions between the different factors. They were also able to identify feedback loops within the system that drive inequities in healthy eating. Through participating in the workshops and constructing the CLD, the policy actors were able to discuss deeply the whole system and begin to identify key points in the system in which there are opportunities to intervene to address inequities in healthy eating.

### Strengths of the study

#### A new way of thinking

This study framed the emergence of inequity in healthy eating as arising from a complex adaptive system and successfully used an expert group collaborative modelling process to build a causal loop diagram for understanding drivers, major feedback loops, and the system structure of inequity in healthy eating. This has not been done as far as we are aware. Previous nutrition related systems research has focussed on the food environment rather than to draw together all of the relevant drivers of diet and nutrition as a complex adaptive system, and has not included an equity focus [51, 52].

#### Participatory processes

By using a collaborative group model building method in the development of the causal loop diagram, the study demonstrated that such a systematic approach was a useful way of engaging key policy actors in developing a broad understanding of systems that affect inequities in healthy eating and ultimately inequities in NCDs. The study was very participatory, and enabled a diverse group of stakeholders to share knowledge and insights about a whole variety of issues relating to healthy eating, in a way that was informative and respectful of differences in views and knowledge. The involvement of senior government officials, key non-government agencies and prominent academics has the potential to enhance the leadership and workforce readiness to push for a comprehensive policy and practice response to inequities in healthy eating. This participatory approach of involving key stakeholders in the process of developing the CLD has been observed by others to help create ownership of the issues raised and help move towards action and solutions [11, 15, 53].

#### Crossing sectors

Almost none of the participants had used systems science approaches previously, and in a relatively short period of time, using the CCM method, deepened their understanding of the wide range of determinants of inequities in healthy eating, and started to think about policies and interventions that they might otherwise not have. Indeed, the complexity of the HE^2^ diagram demonstrates that bringing together the range of experts in the field of healthy eating enabled the identification of many connections between many factors that influence equity in healthy eating. As a group, the experts identified seven sub-systems that are important to address inequities in healthy eating: food supply and environment; housing and the built environment; transport; employment; social protection; health literacy, and food preferences. The HE^2^ CLD provides a useful tool for engaging actors from less ‘traditional’ sectors (e.g. Housing, Social Protection, Employment), which is a critical first step in tackling this complex systems-wide issue of inequities in healthy eating.

#### A model for complex problems

Using a systems approach to understand the drivers of inequities in healthy eating has enabled us to produce a new conceptual model of HE^2^. This visual product, plus the method used to develop it, is likely to be of value to others concerned with complex health and social issues. Not only does the CLD help visualise the different drivers of complex problems, in this case inequities in healthy eating, it also demonstrates the interconnections and feedback loops, which ultimately can help the users identify key points in the system in which to intervene.

### Weaknesses of the study

As with other studies that have used systems science to investigate health issues [14], the resulting CLD is complex, messy and potentially overwhelming. However, visualising the complexity helped the expert group ‘see themselves’ in the potential solutions to inequities in healthy eating and how their actions were connected to many other parts of the system that they would otherwise not have known.

As is the case for all causal loop diagrams [48], the HE^2^ diagram cannot visually represent the distributional effects of the different variables on healthy eating. The HE^2^ diagram represents important variables that impact on inequities in health eating, the structure of these variables, and their relationships, but not the specific degree that these affect an individual or group. However, the diagram can be used in an interpretive manner, noting that while the depicted policy areas and determinants matter for all people, different groups are affected to different degrees. For example, access to affordable public transport is relevant for accessing food for all people, however the degree to which different individuals or groups experience transport as a barrier to healthy eating varies.

Another limitation of the study is the positioning of the boundaries around the system. The boundaries were determined by the interests and knowledge of the expert group and may have differed if the composition of the group was less health prevention focussed and more inclusive of a business perspective or directly involved community organizations engaged in promoting or preserving healthy eating alternatives. The CLD may therefore not be generalizable to other policy actor groups and communities. However, the purpose of this study was to provide a way by which policy actors could begin to engage with, and apply, systems thinking.

While study participants were encouraged to think in an analytical manner about a complex system, the study must be considered descriptive in nature rather than analytical. As a descriptive study, the results are however valuable as a first step in understanding and appreciating the processes that were undertaken to address the research question and provide a platform from which to continue such a body of work.

The method used in this study relies on the knowledge of an assembled group of experts. This brings with it some potential biases and knowledge gaps. Complementing this method could be meta-narrative synthesis mapping exercises, which characterise the literature in complex issues (e.g. inequities in healthy eating) in order to identify areas where published evidence exists or where gaps can be ascertained. For example, Weiler et al [54] undertook such an exercise to examine literature exploring the relationships between food sovereignty, food security, and health equity. Iteration between experts and literature would help overcome knowledge gaps and group biases.

### Implications of the study

This conceptualisation of the drivers of inequities in healthy eating helps demonstrate to a range of policy actors the importance of policy action that tackles the systemic drivers of the availability, affordability, accessibility and acceptability of healthy food compared to unhealthy foods, and that these actions are not confined to the food system or food environment. The identification of seven broad policy domains that affect inequity in healthy eating suggests that whole of government action is needed.

Further work is needed to elicit different stakeholder perspectives, including food industry, social policy and consumer groups, on the issues that affect healthy eating. The group model building methods used in this study provide a relatively straightforward technique that could be used by policy actors to engage communities. This would go some way to helping ensure policy actions respond to people’s needs.

A quantitative system dynamics model based on the HE^2^ diagram could generate further insight into the utility of the model, and enable policy makers to identify the relative impact of different policy actions at various intervention points in the model. Similar work has been done in modelling diabetes [55] and obesity more broadly [56], which could provide examples of data driven models that could be adapted to the social distribution of healthy eating.

In conclusion, this study aimed to organise, from a complex systems perspective, current understandings of how individual and societal level factors interact to create inequities in healthy eating as part of a larger body of work asking the question: ‘What kind of insight can policy actors gain about the causes and potential solutions to inequity in healthy eating using systems science methods?’ The application of the collaborative conceptual modelling systems approach helped answer this through identifying the complexity of the causes of inequities in healthy eating; visually illustrating the system and its structures, and by beginning to identify leverage points for change within the system.
